# Supplementary material for: Wildfire impacts and mitigation strategies among California cannabis producers
Source: PLoS One. 2025 Apr 11;20(4):e0321476. doi: 10.1371/journal.pone.0321476 (PMC11990751; doi:10.1371/journal.pone.0321476)
Supplement: S1 File — Survey: “Wildfire Impacts on California Cannabis Production.” Survey conducted via Qualtrics. (PDF) [file pone.0321476.s001.pdf]

Berkeley
UNIVERSITY OF CALIFORNIA

Wildfire Impacts on California Cannabis Production

Dear Participant,

Thank you for taking the time to complete our survey! This research is a portion of a larger project conducted by the Cannabis Research Center at the University of California, Berkeley, with support provided by a grant from California's Department of Cannabis Control.

An earlier portion of this research highlighted the frequent overlap between cannabis growing regions and areas of high wildfire risk in California. With this state-wide survey, we hope to collect data around the **impacts of wildfire and smoke exposure on outdoor cannabis production** over the past 5 years, as well as **mitigation strategies** employed by farmers around the state.

In the sections that follow, you will be asked questions about your operations, your experiences with wildfire and smoke, any strategies you have employed to navigate these challenges, and some of the broader context of cannabis production in California. Information you provide will **help us to develop policy recommendations, share best practices from growers around California, and inform future research**.

The survey should take approximately **10-20 minutes** to complete. All information you provide will be anonymous (any identifying information will be collected on a separate site in order to maintain anonymity), and data will remain encrypted and secured by the University of California, Berkeley. Your participation in this research is voluntary, and you may withdraw your consent and stop participation at any time.

By continuing, you indicate that you are an adult (18+), involved in cannabis cultivation in the state of California, and agree to participate in this research study. Please print a copy of this page to keep for future reference, then **click "Next" to proceed to the survey**.

1. Operations

First, we would like to learn a little about your operation. This information will give us a sense of geographical variability and response rates across the state. For purposes of this survey, we define 'operation' as your primary cultivation site -- where you have the most plants and/or spend the most time. If you have multiple non-adjacent farm sites about which you would like to share information, you may complete the survey multiple times for each location.

What is your primary role or affiliation at your operation?

- ○ Operation owner
- ○ Farm employee
- ○ Cannabis processing and services
- ○ [ ] Other

When did you begin cultivating cannabis in California?

[▾]

Are you a member of any cannabis cultivator or trade organizations? *(e.g. a county-level growers alliance, or the California Cannabis Industry Association)*

- ○ Yes
- ○ No
- ○ Decline to state

Please indicate which organization(s) below *(you may also decline to state)*.

[ ]

Which of the following cultivation types are present at your operation?

- ☐ Outdoor
- ☐ Indoor

- [ ] Mixed-light
- [ ] ______ Other

Are your operations licensed?

- ○ Yes
- ○ No
- ○ Currently pursuing licensing
- ○ Decline to state

Where is your operation located? *(This refers to your primary cultivation site; if you have multiple non-adjacent farm sites, you may complete the survey multiple times for each location)*

County ______

Zip Code ______

How much land did you have under cannabis cultivation in the last year?

______

Which of the following does your operation produce? *(Note that we are concerned with final consumer products, whether these are processed in-house or by a third party)*

- [ ] Non-manufactured product (e.g., flower, immature plants, seeds, pre-rolls)
- [ ] Manufactured product (e.g., edibles, concentrates, topicals)
- [ ] ______ Other cannabis product
- [ ] ______ Non-cannabis commercial agricultural product

2. Impacts

This section is concerned with grower experiences of fire, smoke, and associated impacts, particularly those over the last four years (2018-2021). Please note that we are also interested in non-impacts, i.e. if you did not *experience something, note this where appropriate. As previously, we define 'operation' here as your primary growing site -- where you have the most plants and/or spend the most time.*

During which of the following years did you cultivate cannabis at your operation?

- ☐ 2021
- ☐ 2020
- ☐ 2019
- ☐ 2018
- ☐ Prior to this period

During which of the following years did you experience cannabis crop loss due to **fire**?

- ☐ I have not experienced fire losses
- ☐ » 2021
- ☐ » 2020
- ☐ » 2019
- ☐ » 2018
- ☐ » Prior to this period

During **2021**, approximately what percentage of your crop was lost due to fire?

- ○ 10% or less
- ○ 25%
- ○ 50%
- ○ 75%
- ○ 100%

During **2020**, approximately what percentage of your crop was lost due to fire?

- ○ 10% or less
- ○ 25%
- ○ 50%
- ○ 75%
- ○ 100%

During **2019**, approximately what percentage of your crop was lost due to fire?

- 10% or less
- 25%
- 50%
- 75%
- 100%

During **2018**, approximately what percentage of your crop was lost due to fire?

- 10% or less
- 25%
- 50%
- 75%
- 100%

During which of the following years did you experience noticeable **smoke** at your operation?

- I have not experienced smoke on my operations
- » 2021
- » 2020
- » 2019
- » 2018
- » Prior to this period

During **2021**, over approximately what percentage of the flowering period was there noticeable smoke on your operations *(including light, non-disruptive smoke days)*?

- None
- 10% or less
- 25%
- 50%
- 75%
- 100% (virtually all)

During **2021**, over approximately what percentage of the flowering period was there **heavy** smoke on your operation *(i.e. during which time a worker might need to wear a mask)*?

- None
- 10% or less
- 25%
- 50%
- 75%
- 100% (virtually all)

During **2020**, over approximately what percentage of the flowering period was there noticeable smoke on your operations *(including light, non-disruptive smoke days)*?

- None
- 10% or less
- 25%
- 50%
- 75%
- 100% (virtually all)

During **2020**, over approximately what percentage of the flowering period was there **heavy smoke** on your operation *(i.e. during which time a worker might need to wear a mask)*?

- None
- 10% or less
- 25%
- 50%
- 75%
- 100% (virtually all)

During **2019**, over approximately what percentage of the flowering period was there noticeable smoke on your operations *(including light, non-disruptive smoke days)*?

- None
- 10% or less

- 25%
- 50%
- 75%
- 100% (virtually all)

During **2019**, over approximately what percentage of the flowering period was there **heavy smoke** on your operation *(i.e. during which time a worker might need to wear a mask)*?

- None
- 10% or less
- 25%
- 50%
- 75%
- 100% (virtually all)

During **2018**, over approximately what percentage of the flowering period was there noticeable smoke on your operations *(including light, non-disruptive smoke days)*?

- None
- 10% or less
- 25%
- 50%
- 75%
- 100% (virtually all)

During **2018**, over approximately what percentage of the flowering period was there **heavy smoke** on your operation *(i.e. during which time a worker might need to wear a mask)*?

- None
- 10% or less
- 25%
- 50%
- 75%
- 100% (virtually all)

During which of the following years did you experience visible **ash** or particulate accumulation on your plants?

- ☐ I have not experienced ash or particulates
- ☐ » 2021
- ☐ » 2020
- ☐ » 2019
- ☐ » 2018
- ☐ » Prior to this period

During **2021**, approximately what percentage of your crop was affected by ash/particulates?

- ○ 10% or less
- ○ 25%
- ○ 50%
- ○ 75%
- ○ 100% (virtually all)

During **2021**, which of the following effects did you experience following smoke exposure and/or ash accumulation?

- ○ Negligible impact on product quality
- ○ Reduction in quality sufficient to reduce value of crop
- ○ Reduction in quality sufficient to render crop unsellable/destroyed
- ○ ______________ Other effect

During **2020**, approximately what percentage of your crop was affected by ash/particulates?

- ○ 10% or less
- ○ 25%
- ○ 50%
- ○ 75%
- ○ 100% (virtually all)

During **2020**, which of the following effects did you experience following smoke exposure and/or ash accumulation?

- ○ Negligible impact on product quality
- ○ Reduction in quality sufficient to reduce value of crop
- ○ Reduction in quality sufficient to render crop unsellable/destroyed
- ○ ______ Other effect

During **2019**, approximately what percentage of your crop was affected by ash/particulates?

- ○ 10% or less
- ○ 25%
- ○ 50%
- ○ 75%
- ○ 100% (virtually all)

During **2019**, which of the following effects did you experience following smoke exposure and/or ash accumulation?

- ○ Negligible impact on product quality
- ○ Reduction in quality sufficient to reduce value of crop
- ○ Reduction in quality sufficient to render crop unsellable/destroyed
- ○ ______ Other effect

During **2018**, approximately what percentage of your crop was affected by ash/particulates?

- ○ 10% or less
- ○ 25%
- ○ 50%
- ○ 75%
- ○ 100% (virtually all)

During **2018**, which of the following effects did you experience following smoke exposure and/or ash accumulation?

- ○ Negligible impact on product quality
- ○ Reduction in quality sufficient to reduce value of crop
- ○ Reduction in quality sufficient to render crop unsellable/destroyed
- ○ ________ Other effect

Have you had any lab-based chemical testing of your product conducted following smoke and/or ash exposure?

- ○ Yes
- ○ No

Please describe the results.

[ ]

Which of the following describe your decision not to test your product?

- ☐ No fire, smoke, or ash occurrence
- ☐ No regulatory requirement for testing
- ☐ Testing unavailable in my area
- ☐ Cost prohibitive
- ☐ Unlicensed grower
- ☐ ________ Other

Which of the following fire-related effects have you experienced on your operations? (*part 1*)

- ☐ Health and safety concerns related to smoke
- ☐ Emergency evacuation notice(s)

- [ ] Power outage(s) during or around periods of fire
- [ ] Use of fire retardant on or in close proximity to crops
- [ ] Road blockage(s) preventing watering and/or harvest of crop

Which of the following fire-related effects have you experienced on your operations? (*part 2*)

- [ ] Loss of seed stock / strains
- [ ] Noticeable crop growth rate decline due to natural light reduction
- [ ] Increase in pests or fungi (e.g. powdery mildew) following fire / smoke
- [ ] Loss or significant damage to structures and/or equipment
- [ ] Supply chain disruptions
- [ ] ________ Other

3. Mitigation

This next section is concerned with grower efforts to avoid and navigate the challenges of fire and smoke, as well as any barriers to mitigation you may have encountered.

Does your operation currently hold fire insurance of any sort?

- ○ Yes
- ○ No

What is your current premium per year? (*in U.S. dollar amount*)

____

Which of the following describes your decision not to hold insurance?

- [ ] Do not see fire as a danger in my area
- [ ] Fire insurance unavailable in my area
- [ ] Cost prohibitive
- [ ] Unlicensed grower
- [ ] ________ Other

Which of the following have you installed and/or are currently in use on your operation to mitigate the effects of wildfire?

- ☐ Air quality trackers / on-farm sensors
- ☐ Alert apps / other fire notification systems (e.g. PulsePoint)
- ☐ Provision of masks / PPE to workers
- ☐ Safety measures / SOPs to reduce risk of ignition (e.g. fire extinguishers)
- ☐ Automatic / drip irrigation systems
- ☐ Water storage on property (e.g., tanks, trucks, ponds)
- ☐ Fuel reduction / brush clearing
- ☐ Roadway improvements to facilitate site access
- ☐ Infrastructure to protect crops (e.g. hoop houses)
- ☐ Spraying / washing particulates off of growing plants
- ☐ Dipping harvested crop in chemical solution
- ☐ ______ Other

Which of these do you see as **most effective** for reducing fire / smoke impacts?

- ○ » Air quality trackers / on-farm sensors
- ○ » Alert apps / other fire notification systems (e.g. PulsePoint)
- ○ » Provision of masks / PPE to workers
- ○ » Safety measures / SOPs to reduce risk of ignition (e.g. fire extinguishers)
- ○ » Automatic / drip irrigation systems
- ○ » Water storage on property (e.g., tanks, trucks, ponds)
- ○ » Fuel reduction / brush clearing
- ○ » Roadway improvements to facilitate site access
- ○ » Infrastructure to protect crops (e.g. hoop houses)
- ○ » Spraying / washing particulates off of growing plants
- ○ » Dipping harvested crop in chemical solution
- ○ ______ » Other

Approximately how much have you / your operations spent in total on fire and smoke mitigation measures?

For each of the following statements, please indicate your agreement or disagreement:

	Strongly disagree	Somewhat disagree	Neither agree nor disagree	Somewhat agree	Strongly agree
Fire and/or smoke has motivated change(s) in **on-farm practices**	○	○	○	○	○
Fire and/or smoke has motivated change(s) in **where** I grow	○	○	○	○	○
Fire and/or smoke has motivated change(s) in the **timing** of planting or harvest	○	○	○	○	○

For each of the following statements, please indicate your agreement or disagreement:

	Strongly disagree	Somewhat disagree	Neither agree nor disagree	Somewhat agree	Strongly agree
Fire and/or smoke has motivated a shift toward **indoor production**	○	○	○	○	○
I am considering **leaving this industry** due to **fire**	○	○	○	○	○
I am considering **leaving this industry** due to **other pressures**	○	○	○	○	○

For each of the following entities, please indicate the degree to which they **hinder** or **help** your wildfire mitigation efforts.

	Greatly hinders	Hinders	Neutral	Helps	Greatly helps	No interaction
California Department of Cannabis Control (CDCC; licensing and testing requirements)	○	○	○	○	○	○
Cal Fire (guidelines and regulations)	○	○	○	○	○	○
County-level fire ordinances and programs	○	○	○	○	○	○

	Greatly hinders	Hinders	Neutral	Helps	Greatly helps	No interaction
Local fire department or community-based fire fighting efforts	○	○	○	○	○	○
California Department of Fish and Wildlife (CDFW)	○	○	○	○	○	○
California Department of Food and Agriculture (CDFA)	○	○	○	○	○	○
US Forest Service (USFS)	○	○	○	○	○	○
California State Water Resources Control Board (SWRCB)	○	○	○	○	○	○
Other ______	○	○	○	○	○	○

4. Final thoughts

Thank you for sticking with us to the end! In this final section, we are interested in your assessment of wildfire and smoke risks in relation to other challenges to cannabis production in California, as well as any final thoughts you'd like to share.

From the following, please select your **top three** sources of concern or insecurity for your operations.

- ☐ Wildfire risk
- ☐ Smoke or ash impacts on crops
- ☐ Pesticide drift / contamination
- ☐ Water availability
- ☐ Land prices
- ☐ State-level regulatory requirements
- ☐ County-level policies
- ☐ Taxes and fees
- ☐ Low prices / price fluctuations
- ☐ Unlicensed growers
- ☐ Legal challenges or federal uncertainties

Is there anything else you'd like to share with us? This could include subjects we haven't covered in the survey, stories you have of dealing with wildfire, or any other

recommendations.

If you are interested in being contacted for additional studies, including possible follow-up interviews, please provide your information here.

Powered by Qualtrics
