# Supplementary material for: Wildfire impacts and mitigation strategies among California cannabis producers
Source: PLoS One. 2025 Apr 11;20(4):e0321476. doi: 10.1371/journal.pone.0321476 (PMC11990751; doi:10.1371/journal.pone.0321476)
Supplement: Attachment — Submitted filename: Response to Reviewers.pdf [file pone.0321476.s002.pdf]

4 December 2024

Dear PLOS ONE Editors,

Thank you for the opportunity to respond to the reviewers' comments on our manuscript, "Wildfire impacts and mitigation strategies among California cannabis producers," and for your patience as we made the necessary changes.

Please find below a response table to the points raised by the academic editor and reviewers. We believe we have adequately responded to each issue but welcome an opportunity to make any additional revisions necessary.

Thank you for your consideration.

Sincerely,

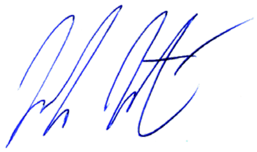

Dr. Jeff Vance Martin
Research Social Scientist
U.S. Forest Service, Pacific Southwest Research Station
1700 Bayview Drive, Arcata, CA 95521-6013
Jeffrey.Martin4@usda.gov

Source	Comment	Response / action
Staff editor	Please ensure that your manuscript meets PLOS ONE's style requirements, including those for file naming. The PLOS ONE style templates can be found at https://journals.plos.org/plosone/s/file?id=wjVg/PLOSOne_formatting_sample_main_body.pdf and https://journals.plos.org/plosone/s/file?id=ba62/PLOSOne_formatting_sample_title_authors_affiliations.pdf	We have attempted to update manuscript to match style requirements, and will happily make any additional changes needed.
Staff editor	In the online submission form, you indicated that, raw data cannot be shared publicly because of anonymity requirements associated with human subjects review. Summarized data can be made available upon request.<br>All PLOS journals now require all data underlying the findings described in their manuscript to be freely available to other researchers, either 1. In a public repository, 2. Within the manuscript itself, or 3. Uploaded as supplementary information.<br>This policy applies to all data except where public deposition would breach compliance with the protocol approved by your research ethics board. If your data cannot be made publicly available for ethical or legal reasons (e.g., public availability would compromise patient privacy), please explain your reasons on resubmission and your exemption request will be escalated for approval.	As previously noted, raw data cannot be shared because it would breach compliance with our human subjects ethics approval; while responses were largely anonymous, many respondents provided potentially identifying information in their qualitative responses and/or in the provision of geographic location data.
Staff editor	Please upload a copy of Figure 5, to which you refer in your text on page 16 and 18. If the figure is no longer to be included as part of the submission please remove all reference to it within the text.	Figure 5 added to figures document; our apologies for this oversight!
Staff editor	Please include captions for your Supporting Information files at the end of your manuscript, and update any in-text citations to match accordingly. Please see our Supporting Information guidelines for more information: http://journals.plos.org/plosone/s/supporting-information.	We have revised supporting information file names, adjusted in-text references, and included Supporting Information information at the end of our manuscript.
Reviewer 1	...you state a couple of times that cannabis is an **important crop**. But it also seems from a couple of statements in the paper as though the highest production does not occur among outdoor growers nor in the Emerald Triangle region. Do you have a sense of how much your survey population contributes to the industry in California as a whole?	We have added additional text exploring the relationship between our respondents and broader dynamics in the industry -- see pp.9-10
Reviewer 1	The **abstract** currently provides too little information. You tells us what you did, why the topic matters, and how you hope your findings could be used. But you don't actually report what you found. Could you include a sentence or two highlighting major findings (e.g., that most growers reported impacts from fire but they don't see them as critical in the face of other impediments to profitability)?	Thank you for this suggestion. We have expanded the abstract to highlight key findings.
Reviewer 1	On p. 10 and again on p. 12, you cite data that shows **wildfires** are increasing in the state, but your statements imply that prior to climate change and fire suppression, the only source of fire on California landscapes was Indigenous burning. That is not correct – fire was part of the landscape before, though probably not frequently in the Emerald Triangle. Also, a third contributor to the current trend is human ignitions, largely due to WUI growth.	Thank you for this correction; we have made edits to the text on pages 2 and 4.

Source	Comment	Response / action
Reviewer 1	I encourage you to look for **repetitions**. For example, the research questions are presented on p. 11 and again at the bottom of p. 13. They only need to appear once.	We have rephrased the repeated instance of our research questions on p.7, but retain them in a modified form as a reminder for the reader. Other instances of repetition we retain for clarity and emphasis given the range of topics discussed.
Reviewer 1	On **p. 14**, you say you focused on growers with outdoor and "**mixed-light**" licenses. However, the **footnote** explaining what "mixed-light" means isn't linked to this first reference but to a second reference on the next page. I suggest moving the footnote to that first reference. Also, please help the reader understand how mixed-light producers differ from indoor producers, since both use greenhouses. (This could go in the footnote as well.)	Footnote has been moved forward to first usage (p.8). Additional text has been added to the footnote to clarify the meaning of mixed light vs. indoor production.
Reviewer 1	A **response rate** of 13% is not at all unexpected, given the time demands on agricultural producers and broad trends in society of not responding to surveys. Still, it means most of the eligible respondents chose not to do so. It would be helpful to provide a sentence or so describing how your respondents might have differed from the overall population, and what biases that might have introduced.	We have added additional text exploring the relationship between our respondents and broader dynamics in the industry -- see pp.9-10.
Reviewer 1	The caption for **Table 1** requires a bit of elaboration. It appears that the question referred to impacts on crop yield and quality, but that's not obvious. (For example, in the figure you referenced at the start of Results, impacts also included human health and incidental effects of fire suppression.)	We have revised the caption for Table 1 in the interest of greater clarity. The aim is to demonstrate relationships between occurence of smoke and ash (left) and impact on crop value (top). Other impacts (e.g., on human health) are shown in Fig 1.
Reviewer 1	...you state a couple of times that cannabis is an important crop. But it also seems from a couple of statements in the paper as though the highest production does not occur among outdoor growers nor in the Emerald Triangle region. Do you have a sense of how much your survey population contributes to the industry in California as a whole?	Figure caption and text revised to remove instances of "observed" in favor of "reported."
Reviewer 1	On p. 23 you refer to a **Figure 5**, but that was not included in my copy of the manuscript.	Fig 5 added to figures document - apologies for this oversight!
Reviewer 1	Also, it is generally thought to be more straightforward to present all results in the Results section rather than waiting for the Discussion. I understand that your manuscript might flow better this way, but I suggest **moving the middle paragraph** of this sub-section on producers' top concerns to the Results section.	We have moved this paragraph (and fig 5) to the end of the results section (pp.19-20), along with our paragraph on mitigation spending (now p.18)
Reviewer 1	One issue that was briefly referenced in your paper, but not specifically discussed, is **water availability**. **Morgan et al. (2021 in Water)** found that water rights concerns in the Emerald Triangle could make it more difficult for growers to get all the water they need. In your study, water storage was seen as less effective. Is it possible that availability affected that response?	We make reference to limitations on water availability in the same paragraph in which we discuss water storage, but we have now added a reference to Morgan, et al. 2021 (p.17).
Reviewer 2	First, there appears to be **several more research questions addressed** in the paper than the two simplistic ones introduced in the Introduction. I suggest the authors identify an over-arching research question that would encompass the multitude of research questions explored through the paper.	Although we covered a range of factors in our survey (and hence reporting in Results), we see these as falling under the broad categories of *impacts* and *responses*. However, these questions appear to us distinct enough to warrant separate research questions as we have organized them here.

Source	Comment	Response / action
Reviewer 2	Second, **theory** is introduced in the discussion but receives only light engagement. I suggest looking at **work in political ecology that engages in discussion about constructed or co-produced vulnerability to wildfires** and then to introduce some of this theory while framing the paper in the introduction. Greater engagement with theory may help to focus your paper a little bit more as it currently seems to just focus on reporting lots of descriptive statistics.	We have added additional text engaging with theory, specifically the political ecology of hazards/disasters. See p.3 (introduction) and at length on pp.5-6 (background)
Reviewer 2	Methods - Include the information about how many **survey responses** you received in this section.	Response rate (previously at the top of Results) has been moved up to the end of our Background and methods (p.7)
Reviewer 2	Methods - It is **unclear about where the narrative responses came from** – was this from the open-ended question included in the survey or from the focus group? Did you use particular open-ended questions to help you understand the patterns that you saw in the descriptive statistics (this would seem logical)? The way it is written now makes it appear that you analyzed all of the open-ended questions together instead of grouping them by topic. The description of your process of your qualitative analysis is pretty vague and confusing. Please clarify in general.	Additional clarifications (and methods citations) have been provided in the methods section (p.9)
Reviewer 2	Results - This section seems to lack focus. It runs on for several pages of **descriptive statistics**. It includes responses to much, much more than the two research questions you identified.	Much of what is described under Results corresponds to the detailed breakdown of our questions provided on pp.7-8. We believe these data provide important information on the types of impacts experienced by producers, along with a preliminary statistical assessment of how such impacts (as well as mitigation practices) may or may not function -- potentially informing future research and/or on-farm and policy interventions.
Reviewer 2	Discussion - This needs to be further **developed and situated** in the literature that you identify.	We have made an effort to reorganize and clarify the discussion, as well as more clearly signpost connections with the literature. Combined with the additions made to the background, we believe this helps clarify the analysis.
Reviewer 2	A paper to look into:<br>**Simon, G.L. and Dooling, S., 2013**. Flame and fortune in California: The material and political dimensions of vulnerability. Global Environmental Change, 23(6), pp. 1410-1423.	Thank you for this recommendation. We have reviewed Simon & Dooling 2013 and incorporated insights into the text (see pp.3, 6)
Reviewer 2	Conclusion - The main finding seems to be that vulnerability is also dependent or co-produced on institutional contexts. This is not a novel finding in itself. I'd like to challenge the authors to **identify how this work may push along the literatures** within which they situate the study.	One major aim of this research was to help lay the groundwork for future research directions, something elaborated in depth in the conclusion (pp.27-28). Part of this includes putting work on cannabis into conversation with literatures on wildfire and community adaptation - we have added some clarification and additional citations to better make this point.
